# Nutritional and Microbial Strategies for Treating Acne, Alopecia, and Atopic Dermatitis

**DOI:** 10.3390/nu16203559

**Published:** 2024-10-20

**Authors:** Alejandro Borrego-Ruiz, Juan J. Borrego

**Affiliations:** 1 Departamento de Psicología Social y de las Organizaciones, Universidad Nacional de Educación a Distancia (UNED), 28040 Madrid, Spain; a.borrego@psi.uned.es; 2 Departamento de Microbiología, Universidad de Málaga, 29071 Málaga, Spain

**Keywords:** skin diseases, acne, alopecia, atopic dermatitis, diet, nutrition, therapeutic tools, probiotics, synbiotics, postbiotics

## Abstract

Background/Objectives: Diet is one of the major determinants of the composition and function of the gut microbiome, and diverse studies have established directional connections between gut microbiome dysbiosis and skin dyshomeostasis. Furthermore, a significant link between the gut and certain skin-related disorders has been reported. This work reviews the mechanisms underlying the relationship between nutritional factors, gut microbiome, and certain skin diseases such as acne vulgaris, alopecia, and atopic dermatitis. In addition, it explores how the modulation of the gut microbiome and human skin through diet and various microbial strategies, including probiotics, synbiotics, postbiotics, and fecal microbiota transplantation, may serve as future treatments for skin diseases, possibly replacing traditional methods such as antibiotic, topical corticosteroid, and laser therapies. Results: The adequate intake of certain foods can promote a balanced gut microbiome, potentially reducing skin inflammation and improving overall skin health, while poor dietary choices may lead to worse outcomes by disrupting gut homeostasis. In this regard, diets high in antioxidants, fiber, and phytonutrients appear to be beneficial for enhancing skin health and preventing associated comorbidities. In addition, the administration of probiotics, synbiotics, and postbiotics in the treatment of cutaneous diseases has been shown to restore skin dyshomeostasis and to improve the symptoms of the reviewed skin conditions. Conclusions: Consuming a healthy, plant-based diet can reduce skin inflammation and enhance overall skin health. Although the application of probiotics, synbiotics, and postbiotics has demonstrated promise in modulating inflammation, enhancing tissue regeneration, and inhibiting pathogenic colonization, further research is required.

## 1. Introduction

Nutritional factors profoundly impact signal transduction pathways that are essential for regulating several systemic processes, with the balance of dietary macronutrients (i.e., carbohydrates, proteins, and lipids), micronutrients (i.e., minerals and vitamins), and bioactive compounds (e.g., resveratrol, tocopherols, tannins, polyphenols) playing a key role in their modulation [1]. In this regard, diet is one of the major determinants of the composition and function of the gut microbiome (GM) [2,3], and the effects derived from this interaction significantly influence overall health, including skin integrity [4].

The outer layer of the skin is composed of keratinocytes that are in direct contact with the external environment and act as a first line of defense to prevent microbial invasion. A second line of protection is formed by antimicrobial peptides (cathelicidin and psoriasin), phagocytes, and innate lymphoid cells [5]. Human skin also provides biochemical conditions such as low pH, high salinity, and xerosis (abnormally dry skin) that prevent many microbial species from successfully colonizing the skin. In addition, the skin harbors a complex microbiota forming the skin microbiome (SM), which exerts microbial competence against allochthonous microorganisms by several mechanisms, including bacteriocin production, phenol-soluble modulins, short-chain fatty acids (SCFAs), antibiotics, and enzymatic activities [6].

Human skin is a true microbial ecosystem, the so-called SM, consisting of bacteria, fungi, and viruses. However, skin varies in its physicochemical and biochemical properties along body sites, resulting in distinct microenvironments within the SM that promote specific microbial populations [7]. The majority of bacteria in the human SM are *Acinetobacter*, *Corynebacterium*, *Cutibacterium*, *Micrococcus*, *Staphylococcus*, and *Streptococcus* [8]. In addition, fungi belonging to the genus *Malassezia*, with the species *M. globosa*, *M. restricta*, and *M. sympodialis*, are the most abundant, although the highest fungal diversity was observed on the feet, colonized by the genera *Aspergillus*, *Cryptococcus*, *Epicoccum*, and *Rhodotorula* [9]. Viruses belonging to the families *Circoviridae*, *Papillomaviridae*, *Polyomaviridae*, and *Poxviridae* have been reported as common members of the SM [10], however, the presence of bacteriophages have also been recently reported [11].

The SM interacts with host epithelial and immune cells in a mutually reinforcing manner. The host provides space and nutrients for the SM, while the SM protects the host from pathogen invasion and modulates the immune system. Interestingly, some commensal microorganisms of the SM can act as opportunists when the SM homeostasis is altered [12]. In this state of dysbiosis, several skin diseases may arise, such as acne vulgaris, atopic dermatitis, psoriasis, chronic wounds, seborrheic dermatitis, rosacea, and alopecia areata [13].

The proposal of the existence of an interaction between the GM and the SM is not new; in fact, together with the brain, these microbiomes form the gut–skin–brain axis [14]. Diverse studies have proven the directional connections between GM dysbiosis and skin dyshomeostasis, with a specific involvement of gut dysbiosis in the pathophysiology of several inflammatory disorders [15,16]. For example, irritable bowel syndrome (IBS) has been associated with skin ulcers, alopecia, erythema nodosum, oral lesions, psoriasis, and pyoderma gangrenosum, while celiac disease has been with dermatitis herpetiformis, alopecia, vitiligo, and psoriasis, and Crohn’s disease with psoriasis and hidradenitis suppurativa [17,18,19]. An association between the gut and skin-related disorders has been reported by Mahmud et al. [4] for the cases of acne vulgaris, rosacea, alopecia, psoriasis, and atopic dermatitis.

Gut dysbiosis possesses the capacity to adversely affect skin function. Microbial metabolites (aromatic amino acids, free phenol, and p-cresol) are released into the blood, reaching the skin and impairing differentiation of epidermal cells, which is reflected in reduced skin hydration and skin barrier integrity [18]. In addition, gut dysbiosis provokes elevated epithelial permeability, which activates effector T cells and triggers the release of inflammatory cytokines that contribute to skin inflammatory disorders via immune and non-immune signaling routes [20]. In contrast, since the year 2010, the positive impact of gut bacteria on skin health has been clearly observed in numerous preclinical and clinical studies. For example, Baba et al. [21] reported that the delivery of *Lacticaseibacillus* (formerly *Lactobacillus*) *helveticus* strain CM4 reduced the extent of dermatitis induced by sodium dodecyl sulfate and the resulting transepidermal water loss (TEWL). A different study demonstrated a substantial improvement in the restoration of skin barrier integrity and also a reduction in markers of reactive skin inflammation after the application of *Lacticaseibacillus paracasei* strain CNCM I-2116 [22]. In a human study, supplementation with the *L. paracasei* strain NCC2461 over two months resulted in reduced skin sensitivity and TEWL, which is an improvement associated with an elevated level of circulating transforming growth factor beta (TGF-β), a cytokine recognized for its beneficial role in maintaining barrier integrity [23].

As the field of dermatology advances, the interaction between the GM and skin homeostasis has emerged as a pivotal focus of investigation for clarifying the pathophysiology of skin conditions and for identifying novel therapeutic targets via the modulation of the microbiome by specific nutrients and bioactive compounds. Therefore, the present work reviews the mechanisms linking nutritional factors, GM, and skin diseases such as acne vulgaris, alopecia, and atopic dermatitis. In addition, it also explores the potential of modulating GM and human skin through diet and various microbial strategies, including probiotics, synbiotics, postbiotics, and fecal microbiota transplantation. In this respect, these approaches are considered promising alternatives and future treatments for skin diseases, with the potential to complement or even replace traditional methods such as antibiotic, topical corticosteroid, and laser therapies.

## 2. Interactions Between GM, Nutrients, and Skin Health

The human GM is formed by a vast collection of bacteria, archaea, fungi, protozoa, and viruses that colonize the intestinal tract [24]. The GM confers essential metabolic and immunological advantages to the host, including the breakdown of indigestible complex polysaccharides, the production of vitamins such as vitamins K and B_12_, the regulation of the immune system by microbial metabolites, and the protection against direct invasion by external pathogens through competitive adhesion to epithelial cells. Additionally, diverse gut microbes and their metabolites, such as retinoic acid and polysaccharide A produced by *Bacteroides fragilis*, *Faecalibacterium prausnitzii*, as well as by *Clostridium* cluster IV and XI bacteria, foster the proliferation of regulatory T cells involved in anti-inflammatory effects [25]. In addition, the microbial metabolites like short-chain fatty acids (SCFAs), particularly butyrate, induce histone deacetylase inhibition, which supports the expansion of regulatory cells active in follicle stem cell differentiation and wound healing [26]. A strong correlation between the GM and skin homeostasis is evidenced by the fact that DNA from gut bacteria was detected in the plasma of individuals with psoriasis [18], suggesting that gut bacteria and microbial metabolites gain access to the bloodstream and accumulate in the skin when the gut barrier is disrupted [18]. In addition, GM appears to affect the SM through SCFAs that determine the bacterial profile of the SM. For example, propionic acid shows an antimicrobial activity against methicillin-resistant *Staphylococcus aureus* [27], while *Staphylococcus epidermidis* and *Cutibacterium* (formerly *Propionibacterium*) *acnes* tolerate broader SCFA shifts than other commensal skin microbiota [26]. The role of nutrients, such as omega-3 fatty acids and polyphenols, in modulating the GM is also pivotal. These nutritional substances can influence microbial diversity and activity, which in turn affects the systemic and local inflammatory responses. Thus, an adequate intake of certain foods can promote a balanced GM, potentially reducing skin inflammation and improving overall skin health, while poor dietary choices may lead to worse outcomes by disrupting gut homeostasis.

### 2.1. Implications of GM and Dietary Factors in Acne Vulgaris

Acne is a chronic inflammatory disorder of the pilosebaceous follicles, predominantly affecting adolescents and young adults. Characterized by excessive sebum production, follicular hyperkeratinization, and inflammation, acne can lead to skin lesions and to a significant psychological impact, since it is associated with increased risk or mental health issues, including anxiety, depression, and diminished self-esteem [28,29,30].

While the GM is just one of a variety of factors influencing acne, it plays a notable role in the condition. Although the precise mechanisms remain still unclear, it is hypothesized that the GM affects immune system regulation. Subjects with acne often exhibit reduced GM diversity, with decreased levels of the members of Bacillota, and increased levels of members of Bacteroidota, particularly *Clostridium*, along with depletion of *Lachnospiraceae* and *Ruminococcaceae* families [31]. Table 1 shows the relationship between GM and acne vulgaris.

**Figure 1:**
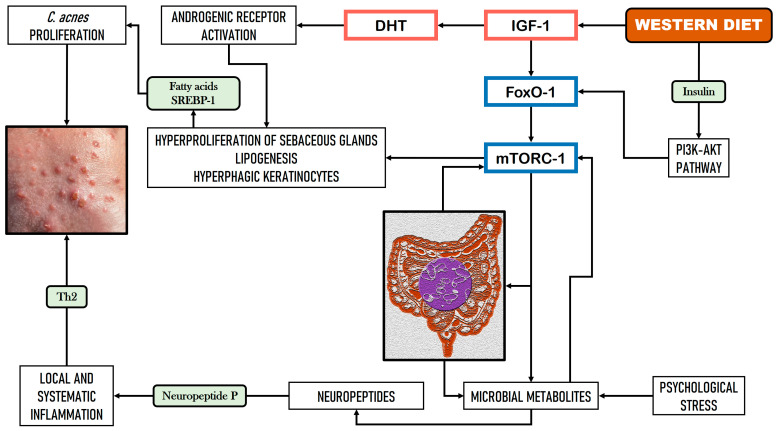
Hypothetical relationship between gut microbiome dysbiosis, the Western diet, and acne (based on Rygula et al. [44] and Melnik et al. [45]).

The Western diet, characterized by high levels of saturated fat and elevated glycemic indices, is closely associated with acne vulgaris [44]. The hypothesized etiology involves dysregulated nutrient signaling, leading to uncontrolled activation of sterol regulatory element-binding protein 1 (SREBP-1) and to heightened synthesis of fatty acids and triglycerides in the sebum, which promotes the development of *C. acnes* [45]. Figure 1 shows a hypothetical scheme of the relationship between GM, the Western diet, and acne vulgaris.

### 2.2. Implications of GM and Dietary Factors in Alopecia

Alopecia refers to the reduction or complete absence of hair in regions where it typically grows. This condition can manifest in various forms, ranging from localized patches to widespread thinning, and can be either transient or lasting. Alopecia affects individuals across all ages and genders and it is identified as a result of diverse underlying causes such as genetic factors, autoimmune disorders, hormonal imbalances, drug use, and fungal or microbial infections. Subjects affected by alopecia may experience considerable emotional distress, which can significantly impact their overall well-being [46,47,48].

The importance and the role of the GM in alopecia are based on a study by Nair et al. [49], which noted that mice treated with antibiotics were safeguarded against the onset of alopecia. This suggests that gut bacteria might compromise the intestinal epithelial barrier, possibly leading to inflammation and autoimmune responses. Thus, this underscores the potential impact of dietary changes on the GM. Another study showed hair growth in alopecic patients treated with fecal microbiota transplantation (FMT) [50]. The link between gut dysbiosis and alopecia areata is suggested by the fact that both share common genes that induce a Th1 response, and lead to the generation of IFN-γ, as IFN-γ signals via a JAK/signal transducer and inductor of transcription (STAT) signal route [51]. Activation of this pathway may provoke atypical proliferation of hair follicle cells and could eventually result in hair loss. Furthermore, dysbiosis of the GM may contribute to other diseases, both locally and systemically [52]. However, a clear link between the potential role of the GM and the pathophysiology of alopecia has not yet been established [35]. Table 1 shows the relationship between GM and various types of alopecia.

**Figure 2:**
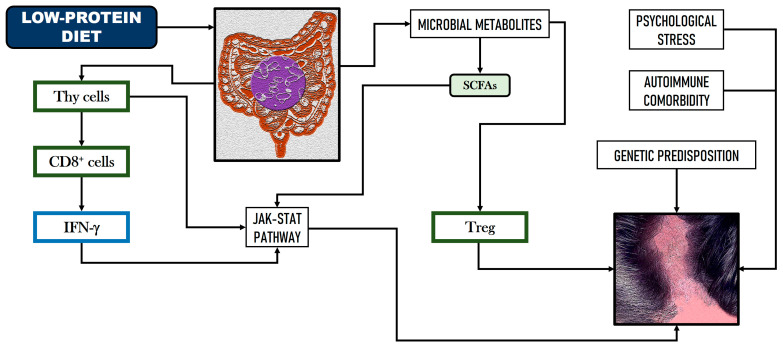
Hypothetical relationship between gut microbiome dysbiosis, low-protein diet, and alopecia vulgaris via the JAK-STAT pathway (based on Simakou et al. [51]).

A nutritional deficiency of proteins, oligoelements, and vitamins can affect hair structure and cause hair loss. Garg and Sangwan [53] investigated in a cross-sectional study the influence of the protein deficiency in alopecia. Almost 90% of alopecic patients exhibited protein deficiency; specifically, 55% of males and 90.9% of females had severe protein deficiency, consuming less than 30 g per day. Scalp biopsies from the low-protein cohort revealed perifollicular chronic inflammation and fibrosis, while those from the high-protein cohort displayed normal follicular structures. Curiously, considering the prevalence of alopecia in the world population and the psychosocial consequences involved, there is limited knowledge on the impact of supplement consumption and its relationship with this condition. However, some dietary changes could also stimulate hair growth in alopecia subjects; for example, a gluten-free dietary pattern boosted hair growth in patients with celiac disease [54]. Figure 2 shows a hypothetical scheme of the relationship between GM, low-protein diet, and alopecia.

### 2.3. Implications of GM and Dietary Factors in Atopic Dermatitis (AD)

AD is a persistent condition marked by intense itching and recurring eczema-like lesions on the skin. It can disrupt sleep and, due to its visible symptoms, may lead to social stigma, diminished self-esteem, isolation, decreased quality of life, and emotional distress. In addition, AD is linked to a higher likelihood of experiencing diverse psychological adverse states such as alexithymia, anxiety, depression, and suicidal thoughts [55,56,57].

GM diversity has been associated with a reduced risk of AD [58]. In addition to the inherent variability of the GM, the interplay between specific gut microbial communities, the immune system, and the balance between the microbiome and the host may determine the onset of AD [59]. Several studies have reported the reduced abundance of *Akkermansia*, *Bifidobacterium*, *Faecalibacterium prausnitzii*, and *Lactobacillus* in individuals with AD compared to healthy controls [38,60]. The GM exerts control over the local and systemic immune system, which can affect peripheral organs such as the skin. The state of GM significantly affects the maturation of naïve T cells into Th1, Th2, Th17, and regulatory T cells (Treg) [61]. Furthermore, several gut genera (*Bacteroides*, *Bifidobacterium*, *Clostridium*, *Lactobacillus*, and *Streptococcus*) and their SCFAs induce the expansion of Treg cells along the body [62]. Gut dysbiosis and the intestinal barrier disruption (leaky gut) have been noted in AD subjects, with a marked reduction in microbial SCFAs. Leaky gut in patients with AD facilitates the entry of toxins, poorly digested foods, and gut microorganisms into the bloodstream, leading to skin inflammation through the induction of Th2, and in turn causing further tissue damage [63]. Table 1 shows the relationship between GM and AD.

**Figure 3:**
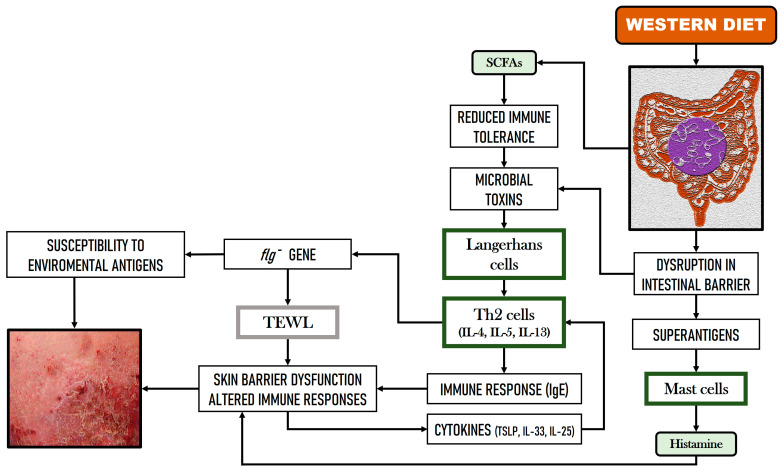
Hypothetical relationship between gut microbiome dysbiosis, the Western diet, and atopic dermatitis (based on Moniaga et al. [62], Kim et al. [63], and Guo et al. [64]).

The Western diet has been linked to the onset of immune-mediated skin conditions like psoriasis and AD. This high-fat diet is thought to induce GM dysbiosis, which in turn alters the balance between Bacillota and Bacteroidota, contributing to inflammation. Guo et al. [64] describe the underlying mechanism of this phenomenon, pointing out that in mice this diet results in a reduced secretion of antimicrobial peptides within the small intestine, changes in the composition of the GM, and Th2-driven inflammation with the subsequent fluctuations in inflammatory cytokine levels. Moreover, a lower intake of fruit, vegetables, and omega-3 fatty acids, coupled with a higher intake of omega-6 fatty acids, has been associated with AD [65]. Figure 3 shows a hypothetical scheme of the relationship between GM, the Western diet, and AD.

## 3. Influence of Healthy Diets on Acne, Alopecia, and Atopic Dermatitis

The influence of diet on the onset of skin disorders is widely acknowledged, with nutritional deficiencies commonly noted as a risk factor for skin disease. In this regard, skin integrity can be adversely affected not only by the lack of certain nutrients, but also by overconsumption and the intake of harmful ingredients [66]. Thus, healthy dietary patterns, such as vegetarian and Mediterranean diets, could play a significant role in modulating the symptoms of various skin disorders, as is the case of acne, alopecia, and atopic dermatitis.

### 3.1. Vegetarian Diets

Vegetarian diets are those primarily based on plant consumption and exclude all forms of animal meat, though they encompass a range of variations, including dietary patterns that incorporate animal derivatives, such as dairy and eggs, as well as strictly plant-based diets. These diets are characterized by an alignment with cultural and ethical motivations, environmental sustainability, and positive correlations with various indicators of physiological health such as a lower risk of cholesterol in blood, type 2 diabetes, syndrome metabolic, ischemic heart disease, or colorectal cancer. However, their impact on mental health outcomes remains inconclusive due to the mixed findings reported [67].

Genetic and environmental factors, including hormonal influences and dietary patterns, play a role in the development of acne [68,69]. The primary mechanisms underlying acne include excessive sebum generation, the overgrowth of *C. acnes*, hyperkeratinization of the pilosebaceous follicles, and inflammatory responses. Among these, IGF-1 is crucial in exacerbating the acne pathogenesis, contributing to the progression and severity of the disease through increased sebum secretion and heightened proliferation of keratinocytes and sebocytes (Figure 1). In addition, IGF-1 is closely related to androgen levels and to hyperinsulinemia [70].

Previous studies have shown that the vegetarian diet may influence the management and prevention of acne, although further research is required to determine whether a plant-based diet can prevent acne entirely, achieve complete control, or merely contribute to standard pharmacological treatments [71]. However, several studies have established the existence of a link between chocolate and milk intake and the onset of acne [72]. Another study also investigated the association between dairy ingestion and acne, finding that whole milk consumption was related to cases of moderate to severe acne, while this association was slightly weaker for low-fat milk [73]. Although the specific mechanisms through which the GM may affect acne development remain unclear, a diet high in fats or in foods with a high glycemic index could affect the gut microbiota, leading to increased intestinal permeability and potentially exacerbating the symptoms of acne [74]. Daily glycemic load intake was positively correlated with both the onset and severity of acne [75]. Therefore, it has been suggested that diets without dairy products and with a low glycemic index may be beneficial for patients with acne. For example, a vegan diet seems to reduce the incidence of acne [76]. This outcome may be attributed to the isoflavones and phytoestrogens present in this type of diet, which counteract sebum production caused by androgens and also reduce the dihydrotestosterone levels [77,78]. Additionally, these compounds might offer protective benefits through their anti-inflammatory properties [79].

Vegetarian diets have been demonstrated to reduce the risk of several types of alopecia [51,54], although these findings are based on classic studies. On the other hand, these diets have been associated with deficiencies in essential nutrients such as iron and B-complex vitamins [80], and also with reduced amounts of the oligoelements iron and zinc, and of the vitamins niacin and biotin, which can affect hair structure thereby causing hair loss [81]. Other vitamins such as D and A activate the hair follicle cycle, and vitamin A specifically stimulates hair follicle stem cells and participates in the hair growth [81]. Curiously, considering the large impact of alopecia in the world population and the psychosocial consequences involved, there is insufficient evidence regarding the effect of supplement intake and its relationship with alopecia. However, certain dietary changes may also promote hair growth in alopecia subjects, as following a diet free of gluten might be effective in achieving this outcome [54]. Interestingly, in a case-control study of 354 alopecic patients, Lai et al. [82] found that the frequent consumption of soybean may be protective against moderate to severe alopecia. In a retrospective study, English and Barazesh [83] concluded that plant-based diets were associated with favorable self-reported changes in hair conditions compared to alopecic patients following a Western diet.

The etiology of AD is multifactorial, involving a combination of genetic and environmental factors alongside immunologic activity. AD is a Th2-driven chronic inflammatory disorder often linked with other distinct atopic conditions, including asthma, allergic rhinitis, and specific food allergies [84]. The pathogenesis of AD involves apoptosis, dysregulated immune responses, fluctuations in the SM, the presence of eosinophils and T lymphocytes, and IgE sensitization. These factors collectively lead to the deterioration of the skin’s stratum corneum and the epidermal barrier it forms [63]. Currently, plant-based diets are considered an adjunct therapeutic alternative for AD. Although traditional studies have reported improvements in AD among patients following a vegetarian diet [85,86], a recent cross-sectional study examined the association between AD in adults and various lifestyle aspects, including alcohol use, dietary choices, obesity, physical activity, stress levels, and sleep patterns. It was observed that neither vegetarian nor vegan diets had any correlation with the presence or severity of AD [87]. A plant-based diet may effectively foster a diverse community of beneficial microorganisms that contribute to optimal gut and skin health [88,89]. High-fat, low-fiber diets can disrupt the GM, potentially triggering an inflammatory response similar to that observed in AD, by decreasing the generation of anti-inflammatory metabolites, particularly SCFAs [26,90]. In contrast, a diet abundant in fruit and vegetables might positively impact AD due to the high flavonoid content, which is regarded as providing substantial antioxidant and anti-inflammatory benefits [91]. Moreover, the modulation of immune responses by dietary fatty acids suggests that a high intake of fats from processed foods could influence the pathophysiology of AD [79].

### 3.2. Mediterranean Diet

A Mediterranean diet is predominantly plant-based, incorporating moderate amounts of fish and poultry, and limited dairy. While contemporary variations may include increased red meat and processed foods, the diet fundamentally emphasizes plant-derived nutrients and healthy fats. This dietary pattern has been consistently associated with improved health outcomes and enhanced longevity, which has contributed to its widespread global recognition [2,92].

A Mediterranean diet is distinguished by its high intake of polyphenols and by its potent anti-inflammatory and antioxidant effects [93]. Long-term prospective trials have pointed out that an adherence to the Mediterranean diet is associated with a reduction in cardiovascular incidents [94]. Furthermore, this dietary pattern has been correlated with improved overall well-being and decreased prevalence of anxiety and depressive states [2,95]. Several studies have established the impact of the Mediterranean diet on the acne. For example, it has been found that lower acne severity in Mediterranean diet consumers, and, in turn, acne patients, presented lower adherence to this diet [96]. The association of acne with a Mediterranean diet has been examined in a French case-control study that identified a linear relationship and an inverse correlation between adherence to the Mediterranean diet and the severity of acne [97]. Another study suggested the existence of a link between the Mediterranean diet and lower levels of IGF-1, a pivotal molecule involved in the pathophysiology of acne [98].

The Mediterranean diet, abundant in raw vegetables, fresh herbs, and isoflavone-rich soy products, provides anti-inflammatory nutrients that could promote hair health and stimulate growth in cases of androgenic alopecia [54]. This diet or its components, such as lycopene-rich ingredients, has been proposed as a good option for its incorporation in natural therapeutics into hair growth regimens [99]. However, only a few studies have related the Mediterranean diet to several types of alopecia. Fortes et al. [100] suggested a reduction in the risk of androgenetic alopecia on 104 male consumers of a Mediterranean diet (fresh herbs and salads). However, recently, Moreno-Arrones et al. [35] reported that a modified Mediterranean diet appears to have not beneficial effects in 20 cases of alopecia areata (universalis and totalis).

Regarding the relationship between a Mediterranean diet and AD, contradictory results have been reported. In this respect, a comprehensive national multicenter study identified a correlation between adherence to the Mediterranean diet and reduced prevalence of asthma, allergic rhinitis, and AD [101]. Nevertheless, this finding was not validated in a subsequent prospective observational study, which did not support the hypothesis that the Mediterranean diet during pregnancy provides protective effects against the onset of AD in childhood [102].

## 4. Microbial Therapeutic Tools for Skin Diseases

A probiotic is defined as viable active microorganisms administered regularly and in sufficient quantities, providing health benefits to the host. Probiotics promote a balanced intestinal bacterial community, regenerate intestinal mucosal cells, stimulate the vagus nerve, and maintain a healthy immune system [103]. Swanson et al. [104] redefined a symbiotic as a combination of live microorganisms and substrates (prebiotics) that are selectively utilized by commensal microorganisms, thereby conferring a health benefit on the host. The term “postbiotic” includes those non-living microorganisms (inactivated-microorganisms or their components or products) that promote or preserve health [105]. Alternative associated terms have been employed as well, including “paraprobiotics”, “parapsychobiotics”, “ghost probiotics”, “metabiotics”, “tyndallized probiotics”, and “bacterial lysates” [105].

These potential therapeutic tools (probiotics, synbiotics, and postbiotics) for skin diseases may be applied topically or ingested systemically. Topical use of these tools can modulate the skin’s innate defense mechanisms and enhance the synthesis of antimicrobial peptides, thereby promoting skin health [106]. In addition, probiotics can endure and effectively colonize the skin [107], induce keratinocytes and sebocytes to generate antimicrobial metabolites [108], compete with pathogens for keratinocyte receptors [109], and create a synergistic impact that enhances the balance of skin microbial populations [110]. On the other hand, inflammatory skin diseases are often associated with an imbalanced GM, therefore, modifying this GM through probiotic administration may represent a promising avenue for improving skin health through its modulatory effect on the immune system (stimulating IL-10 release and increasing peripheral Tregs) [111]. In addition, these tools have the capacity to activate beneficial hypothalamic hormones, preserve epithelial integrity, and improve immune tolerance [112,113].

### 4.1. Impact of Microbial Therapeutics on Acne

Small intestinal bacterial overgrowth (SIBO) has been associated with the production of noxious metabolites and with the impairment of enterocytes in the small intestine, which may result in heightened intestinal permeability [114]. Psychological stress and impaired small intestinal transit can exacerbate SIBO and disrupt intestinal barriers [115], with evidence showing substantially elevated SIBO levels in subjects with acne rosacea compared to those without it [116]. Clinical trials have investigated the effect of probiotics on acne mitigation. The intake of *Lactobacillus*-fermented dairy foods, especially when paired with lactoferrin, has shown beneficial outcomes on clinical symptoms in individuals with acne patients [117]. Additionally, probiotics have been linked to a reduction in inflammatory indicators and oxidative stress in subjects with acne, particularly in decreasing inflammatory cytokines such as IL-1α [118]. Research indicates that *Lactobacillus acidophilus*, *L. delbrueckii* subsp. *bulgaricus*, and *Bifidobacterium bifidum* can markedly diminish acne lesions after a 12-week period when administered in conjunction with minocycline [119]. Furthermore, probiotics have been explored for their potential to influence insulin-signaling genes, a critical element in acne pathogenesis. Fabborcini et al. [120] showed that patients with rosacea had significant acne reduction after 12 weeks of oral *Lacticaseibacillus rhamnosus* strain SP-1. Consumption of this probiotic might decrease the insulin-like growth factor 1 (IGF-1) levels, while increasing forkhead box protein O1 (FoxO-1) expression in the skin [120]. In essence, the investigation of the effects of probiotics on skin diseases shows encouraging potential for the treatment of acne. The mechanisms encompass modulation of the immune system, interactions between the gut and the skin, and the rebalancing of the GM. Further and more extensive research is necessary to thoroughly comprehend the long-term impact and optimal delivery methods for these advantageous microorganisms.

Recent human interventions on the role of microbial therapeutics in acne vulgaris are shown in Table 2. Majeed et al. [121] compared a postbiotic with benzoyl peroxide in the treatment of 68 acne patients. The postbiotic was safe, pH stable, thermoresistant, and had antimicrobial activity against *C. acnes* due to the inhibition of 5-α reductase activity. Cui et al. [122] prepared a lotion containing a probiotic that improved acne lesions and decreased the TEWL and sebum generation after four weeks of treatment compared to the baseline. Ma’or et al. [123] reported that the synbiotic skin care application improved the SM status and reduced the skin pathologies. Podrini et al. [124] formulated an anti-acne serum with *Lactiplantibacillus* (formerly *Lactobacillus*) *plantarum* that mimicked lipid overproduction, had anti-inflammatory properties, and ameliorated acne disease skin models. Finally, Rybak et al. [125] noted that probiotic supplementation resulted in a decrease in the facial sebum excretion rate and in an increase in overall TEWL. Participants with acne showed improvements in total, non-inflammatory, and inflammatory lesion counts, as well as improvements in markers of intestinal permeability.

### 4.2. Impact of Microbial Therapeutics on Alopecia

Diverse preclinical studies have reported on the role of probiotics in alopecia. In a study performed by Levkovich et al. [126], mice supplemented with *Limosilactobacillus* (formerly *Lactobacillus*) *reuteri* showed enhanced dermal thickness, heightened folliculogenesis, and increased sebocyte generation. In a different study with rodents, Horii et al. [127] observed that oral supplementation of heat-inactivated *Levilactobacillus* (formerly *Lactobacillus*) *brevis* strain SBC8803 led to diminished cutaneous arterial sympathetic nerve activity, to a reduction in TEWL, and to an enhancement in cutaneous blood circulation. These effects may be attributed to increased serotonin secretion from intestinal enterochromaffin cells and the consequent stimulation of parasympathetic pathways. In a classic human clinical study, Ogawa et al. [128] administered heat-killed *L. brevis* strain SBC8803 oral supplements for 12 weeks. Human subjects exhibited a marked reduction in TEWL and a significant enhancement in corneal hydration.

Recent human interventions in the role of microbial therapeutics in alopecia are shown in Table 3. Two studies used the FMT to ameliorate gastrointestinal infections, Crohn’s disease, and colorectal cancer, resulting in an increase in hair growth in several areas of the patient’s body [50,129]. Later, several studies focused on the use of probiotics, synbiotics, and postbiotics for the treatment of alopecic disorders [130,131,132,133]. The overall results indicated an improvement in hair growth and also an induced modification in GM.

### 4.3. Impact of Microbial Therapeutics on Atopic Dermatitis

In AD, there are skin barrier dysfunction induced by IL-17 and IL-22, immune dysregulation, and shifts in the SM composition [63]. The modulation of GM by promoting *Bifidobacterium* and *Lactobacillus* species produces bioactive conjugated linoleic acid isomers that modulate the Th2 response with a reduction of pro-inflammatory cytokines (IL-4, IL-5, IL-6, IL-13, TNF-α, IFN-γ, and high-sensitivity C-reactive protein) [134]. In addition, probiotics induce the production of anti-inflammatory cytokines such as IL-10 and TGF-β [134], and inhibit the differentiation of mature dendritic cells and the conversion of T cells to Th2 cells [135]. Recent human interventions in the role of microbial therapeutics in AD are shown in Table 4.

Several interventions involving microbial products have been employed to manage AD, utilizing both oral [136,138,139,141,142,143,144,146,147,148,149] and topical [137,140,145] applications. Most of the treatments included probiotics [136,137,138,141,143,144,146,147], while synbiotics were also used in a variety of studies [139,142,145,149]. In addition, other interventions applied postbiotics [137], microbiome transplantation [140], and a mixture of probiotics, prebiotics, and postbiotics [148] in order to assess their collective efficacy. The results across different procedures generally indicated beneficial effects on AD symptoms. However, the study by Dissanayake et al. [142] found no significant impact from either the skin care regimen or the symbiotic treatment on reducing AD development or food allergen sensitivity by the age of 1 year.

## 5. Discussion

In this review, we have examined the link between GM and SM, as well as the influence of diverse microbial therapeutic strategies (e.g., probiotics, prebiotics, synbiotics, postbiotics, and FMT) on the pathophysiology of acne vulgaris, alopecia, and AD. It is clear that skin dyshomeostasis may induce pathological skin disorders, which are prevalent conditions affecting populations globally [150,151]. In addition, the existence of a gut–skin–brain axis has been demonstrated, communicating between these organs through multiple pathways, including gut microbial metabolites, the neuroendocrine system, diet, and the central nervous system [18]. GM synthesizes a wide variety of substances that can potentially alter SM homeostasis. For example: SCFAs, which exert anti-inflammatory effects on the skin [152]; acetylcholine, which affects the barrier functions [153]; gamma-aminobutyric acid, which inhibits itch [154]; dopamine, which causes inhibition of hair growth [155]; and serotonin, which is involved in melatonin synthesis [156].

In the present work, we have also reviewed the role of healthy diets, such as plant-based and Mediterranean diets [157,158], on the skin’s health. Several studies have provided an important understanding of the connection between diet and skin health, however, it is necessary to be aware of the limitations of these studies [66]. For example, the responses to the diets varied significantly among the studies, and the bias and confounding variables did not allow accurate results to be obtained. Nevertheless, it is clear that healthy diets and eating specific nutrients are beneficial for various skin conditions, such as acne [71,76,79], alopecia [54,83], and AD [91], with vegetarian diets being better than the Mediterranean-style eating pattern. Diets high in antioxidants, fiber, and phytonutrients appear to be beneficial for enhancing skin health and for preventing associated comorbid conditions. In addition, the very low-calorie ketogenic diet has been suggested to be effective at reducing the exacerbation of clinical manifestations of acne disease [159,160,161]. The inclusion of prebiotics via dietary habits might also contribute to keep an equilibrated GM, potentially ameliorating inflammatory skin disorders. Customized dietary strategies designed to meet individual requirements and skin conditions will be desired in the future. For example, for acne vulgaris, the personalized diet will need to include fiber-rich foods (oatmeal, beans, apples, and carrots), omega-3 fatty acids, and nuts. In the case of a specific diet for alopecia areata, the most appropriate components are raw vegetables (gluten-free), whole grains, legumes, and fruits (citrus fruits, cherries, apples, berries, and grapes). Finally, in the case of AD, the main components of the personalized diet must be composed of a dietary pattern free of eggs (for infants) and of a supplement based on probiotics.

**Figure 4:**
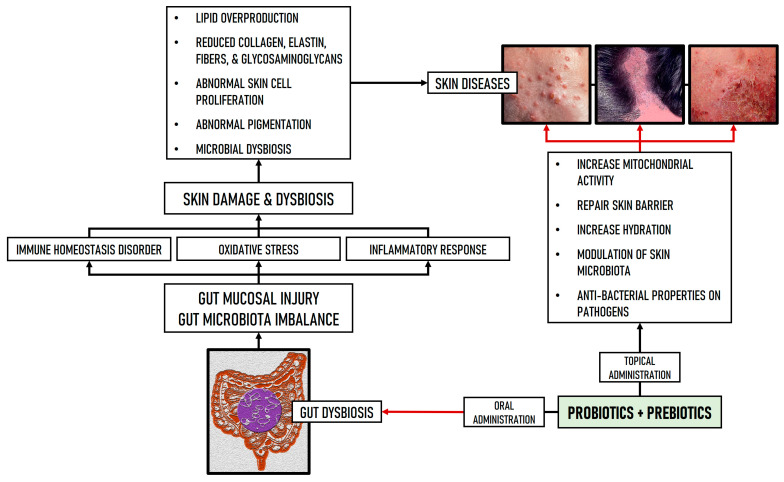
Mechanisms of probiotics and prebiotics involved in the improvement of skin diseases (red arrows represent inhibition processes). Probiotics can restore gut homeostasis by improving GM dysbiosis and repairing intestinal mucosal damage. Additionally, probiotics mitigate skin damage by inhibiting oxidative stress, inflammatory response, and immune homeostasis. Prebiotics, in turn, enhance probiotics growth.

GM dysbiosis is often reflected in the appearance of skin disorders [17,19]. Therefore, the use of probiotics, synbiotics, and postbiotics, both orally and topically, in the treatment of cutaneous diseases, has been proposed to restore skin dyshomeostasis [162]. In addition to the safety limitations of the oral use of probiotics [163], further studies are needed to demonstrate the efficacy, mechanisms of action, and especially the safety of the topical use of probiotics as dermatologic therapy and skin care [164]. Probiotics, as live microorganisms, present more sensitivity to temperature, humidity, and environmental conditions, thus, several factors can affect the quality of probiotics during storage or delivery [165]. To address these concerns, some alternatives have recently been proposed such as synbiotics, postbiotics, microbiome transplantation [123,140,166], or probiotic hydrogels [167] that immobilize bacteria and protect them from immunological reactions on the skin. Figure 4 shows the mechanisms and pathways related to probiotics and prebiotics administration, which are involved in gut dysbiosis and skin diseases.

Beyond the central focus of the research, highlighting the implications of the three reviewed skin diseases on mental health remains pivotal, as these conditions significantly impact the psychological well-being of affected individuals [151]. One of the distinctive and shared characteristics of acne, alopecia, and AD is precisely their high visibility, which is an aspect that highly exacerbates its potential stigmatizing capacity [168]. Indeed, to be affected by any of these conditions potentially contributes to the increased risk of social rejection, bullying, and victimization [169], which are phenomena closely linked to specific emotions, such as shame and humiliation, that have also been related to skin diseases [170,171]. These psychosocial effects can, in turn, lead to severe mental health consequences, such as diminished self-esteem and internalizing disorders, frequently driving individuals to engage in desperate measures to conceal or alleviate their conditions, which could have counterproductive effects on both their mental health and skin health. For instance, individuals with acne may resort to self-injury by manually extracting sebum or pus from pimples, provoking wounds and scarring; those with alopecia might use harmful products that promise hair growth, and attempt to cover bald patches with markers or other unseemly alternatives; while subjects with AD may apply occlusive makeup or wear excessive clothing to cover affected areas. On the other hand, anxiety, depression, and frustration generated by skin diseases may influence those affected to adopt unhealthy dietary patterns, which, in turn, could worsen the skin conditions themselves. Therefore, these factors underscore the imperative necessity to explore non-harmful therapies, particularly nutritional or lifestyle interventions, that can mitigate as far as possible the symptoms associated with these skin disorders. In this respect, treatment with probiotics and synbiotics could not only help to mitigate the severity of skin conditions, but also alleviate stress, anxiety, and resulting depression [172].

## 6. Conclusions

Obtaining a deeper understanding of the complex and diverse roles of the gut–skin axis opens new research horizons. Consuming a healthy diet, mainly plant-based, that is rich in antioxidants, fiber, and phytonutrients, contributes to a balanced GM, which can reduce skin inflammation and enhance overall skin health. Developing techniques to trace the movement of cytokines and compounds induced by probiotics from the gut to the skin can provide valuable insights into their mechanisms of action. Additionally, it is essential to identify microbial products or potential therapeutic tools that positively impact the gut–skin axis. Regarding this, although the application of probiotics, synbiotics, and postbiotics has demonstrated promise in modulating inflammation, enhancing tissue regeneration, and inhibiting pathogenic colonization, further research and clinical trials are necessary to fully realize the therapeutic potential of these agents for addressing skin diseases.

## Figures and Tables

**Table 1:** Relationship between gut microbiome (GM) and skin diseases (acne, alopecia, and atopic dermatitis).

Study/Country	Skin Disease	Sequencing Method	Changes in GM
Deng et al. [31]/China	Acne vulgaris.	16S rRNA and NGS sequencing.	Increased: Bacteroidota members. Decreased: α-diversity, *Lachnospiraceae* and *Ruminococcaceae*.
Yan et al. [32]/China	Acne vulgaris.	16S rRNA sequencing.	Increased: Pseudomonadota members. Decreased: *Allobaculum*, *Bifidobacterium*, *Butyricicoccus*, *Coprobacillus*, and *Lactobacillus*.
Thompson et al. [33]/USA	Acne.	16S rRNA sequencing.	Increased: Bacteroidota members. Decreased: *Lactobacillus iners*, *Lacticaseibacillus zeae* and *Bifidobacterium animalis*.
Huang et al. [34]/China	Acne vulgaris.	V3-V4 regions of 16S rRNA sequencing.	Increased (males): Bacillota members. Decreased (males): *Aerococcus*, *Alkaliphilus*, *Bacillus*, *Bilophila*, *Blautia*, *Butyricicoccus*, *Carnobacterium*, *Enterococcus*, *Exiguobacterium*, *Faecalibacterium*, *Gemmiger*, *Lachnospiraceae incertae sedis*, *Lactococcus*, *Lysinibacillus*, *Oceanobacillus*, *Paenibacillus*, *Pseudomonas* and *Ruminococcus.* Increased (females): *Clostridium sensu stricto*. Decreased (females): *Odoribacter* and *Oscillibacter*.
Moreno-Arrones et al. [35]/Spain	Alopecia universalis.	16S rRNA sequencing.	Increased: *Bacteroides eggerthii*, *Clostridiales vadin* BB60 group, *Erysipelotrichaceae*, *Holdemania filiformis*, *Lachnospiraceae*, *Parabacteroides distasonis*, and *P. johnsonii*.
Juhasz et al. [36]/USA	Alopecia areata.	16S rRNA and ITS sequencing.	Decreased: Clostridia group.
Brzychcy et al. [37]/Poland	Alopecia areata.	V3-V4 regions of 16S rRNA sequencing.	Increased: *Bifidobacterium*, *Eubacterium*, *Lachnoclostridium*, and *Streptococcus.*
Song et al. [38]/Korea	Atopic dermatitis.	16S rRNA sequencing.	Increased: *Faecalibacterium prausnitzii*.
Nylund et al. [39]/Finland	Atopic dermatitis.	16S rRNA microarray.	Increased: *Coprococcus eutactus*. Decreased: Microbial diversity.
Lee et al. [40]/Korea	Atopic dermatitis.	16S rRNA pyrosequencing.	Increased: Clostridia group.
Reddel et al. [41]/Italy	Atopic dermatitis.	16S rRNA metagenomic analysis.	Increased: *Bacteroides*, *Faecalibacterium*, *Oscillospira*, *Parabacteroides* and *Sutterella.* Decreased: *Bifidobacterium*, *Blautia*, *Coprococcus*, *Eubacterium*, and *Propionibacterium*.
Park et al. [42]/Korea	Atopic dermatitis.	Whole metagenomic sequencing.	Increased: *Streptococcus*. Decreased: *Akkermansia* and *Clostridium*.
Nekrasova et al. [43]/Russia	Atopic dermatitis.	16S rRNA and NGS sequencing.	Increased: Members of the families *Erysipelotrichaceae*, *Pasteurellaceae*, *Ruminococcaceae* and *Sutterellaceae*. Decreased: Members of the family *Barnesiellaceae.*

**Table 2:** Human interventions on the role of microbial therapeutics in acne vulgaris.

Study/Country	Intervention	Treatment	Via of Administration	Clinical Assessment	Main Findings
Fabbrocini et al. [120]/Italy	*N* = 20 acne patients (average age: 33.7). 12 weeks. A pilot randomized, double-blind, placebo controlled study.	Probiotic: *Lacticaseibacillus rhamnosus* SP-1.	Oral.	IGA-AS.	Probiotic normalized skin expression of genes involved in insulin signaling and improved the appearance of adult acne.
Majeed et al. [121]/India	*N* = 68 acne patients (age: 18–25). Four weeks. Pilot study.	Postbiotic: LactoSporin (extracellular filtrate of *Bacillus coagulans* MTCC 5856).	Topic.	VISIA.	Efficacy of LactoSporin was similar to that of benzoyl peroxide in reducing sebaceous secretion and the greasy nature of the skin. Presented anti-microbial activity against *C. acnes*.
Cui et al. [122]/China	*N* = 22 acne patients (age: >16). Twenty-one days. Randomized open labeled clinical study.	Postbiotic: Heat-inactivated *Lactiplantibacillus plantarum* VH Probi E15.	Topic.	GAAS.	Anti-acne lotion significantly improvement in acne lesions after 4 weeks of treatment.
Ma’or et al. [123]/Israel	*N* = 31 women volunteers (average age: 23). Four weeks. Clinical trial.	Synbiotic: Spores of *Bacillus subtilis*, *B. licheniformis*, *B. megaterium*, and *B. amyloliquefaciens* (probiotics), with inulin (prebiotic).	Topic.	Acne-QoL, IGA-AS, RBX.	Significant reduction in IGA dermatologist score of acne severity. Reduction in the number of acne lesions. Improved Acne-QoL scores.
Podrini et al. [124]/Italy	Skin cell cultures (skin models) of 8-mm diameter.	Probiotic: *Lactiplantibacillus plantarum* LP01.	Topic.	In vitro study.	Anti-acne serum with the probiotic mimics the over-production of lipids, has anti-inflammatory properties, and improves acne symptoms.
Rybak et al. [125]/USA	*N* = 25 acne patients. Four weeks. Prospective, single-blinded, placebo-controlled study.	Probiotics: Spores of *Bacillus subtilis* HU58, *B. licheniformis*, *B. clausii*, *B. indicus* HU36, and *B. coagulans.*	Oral.	GAAS.	Probiotic supplementation increased the circulating acetate/propionate ratio, and resulted in a decreasing facial sebum and increased TEWL. Patients with acne showed improvement in total lesions and non-inflammatory lesions.

Acne-QoL: Acne Quality of Life Index Questionnaire; GAAS: Global Acne Assessment Scale; IGA-AS: Investigator Global Assessment-Acne Severity; RBX: Redness from Acne Lesions; VISIA: Facial Image Analysis.

**Table 3:** Human interventions in the role of microbial therapeutics in alopecia.

Study/Country	Intervention	Treatment	Via of Administration	Main Findings
Rebello et al. [50]/USA	*N* = 1 alopecic male of 34 years-old with infection of *Clostridioides difficile*. N = 1 alopecic male of 20 years-old with Crohn’s disease. Case report.	Fecal Microbiota Transplantation (FMT).	Colonoscopy.	At follow-up of 8 weeks, hair growth on head, face, and arms of patient 1. After FMT, the patient had regrowth of hair in several sites of his body.
Xie et al. [129]/China	*N* = 1 alopecic male of 86 years-old with a sigmoid colon carcinoma with diarrhea for 6 months. Case report.	Fecal Microbiota Transplantation (FMT).	Colonoscopy.	Diarrhea symptoms remarkably improved one month after FMT. New hair growth in the affected region of his scalp.
Park et al. [130]/Korea	*N* = 46 patients with alopecia (average age: 46.5 [males] and 44.2 [females]). Four weeks. Clinical pilot study.	Synbiotic: *Leuconostoc holzapfelii*, *Leuconostoc mesenteroides*, and *Latilactobacillus* (formerly *Lactobacillus*) *sakei* (probiotics), with Hasou extract + Korean black soybean extract (prebiotics).	Oral.	Synbiotic promoted hair growth and reversed loss without adverse gastrointestinal effects.
Rinaldi et al. [131]/Italy	*N* = 160 patients with alopecia areata (age: 18–60). Three months. Randomized double-blinded parallel-group study.	Postbiotic: Plantaricin A and *Apilactobacillus* (formerly *Lactobacillus*) *kunkeei* ferment product.	Topic.	Efficacy of bioactive peptide on the severity of alopecia compared to control group.
Liang et al. [132]/Taiwan	*N* = 50 adults with hair loss (age: >20). Twelve weeks. Double-blind, placebo-controlled study.	Probiotic: *Lactiplantibacillus plantarum* TC1999.	Oral.	Probiotic increased mitochondrial activity and hair cell growth. Improved gut microbiome.
Navarro-Belmonte et al. [133]/Spain	*N* = 26 alopecic patients (age: >18). Twenty-four weeks. Randomized, double-blind, two-arms, pilot clinical trial.	Probiotics: *Lacticaseibacillus rhamnosus* CECT 30580 and *Bifidobacterium longum* CECT 30616.	Oral.	Probiotic mixture appeared to improve the course of alopecia areata. Skin microbiota of scalp lesions was modified after probiotic treatment.

**Table 4:** Human interventions on the role of microbial therapeutics in AD.

Study/Country	Intervention	Treatment	Via of Administration	Clinical Assessment	Main Findings
Wang and Wang [136]/Taiwan	*N* = 220 AD patients (age: 8–18). Four months. Prospective randomized, double-blind, placebo-controlled study.	Probiotics: *Lacticaseibacillus paracasei* and *Limosilactobacillus fermentum*.	Oral.	SCORAD, FDLQI, CDLQI.	Children who received probiotic mixture showed lower SCORAD scores compared to the placebo group.
Blanchet-Rethore et al. [137]/Germany	*N* = 31 AD patients. Three weeks. Open label multicenter study.	Postbiotic: Heat-treated *Lactobacillus johnsonii* NCC 533.	Topic.	SCORAD.	The application of the lotion with the postbiotic to the lesions of patients with AD controlled *Staphylococcus aureus* colonization and was associated with local clinical improvement.
Wu et al. [138]/Taiwan	*N* = 30 AD patients (age: 4–48 months). Eight weeks. Two center, randomized, double-blind, placebo controlled study.	Probiotic: *Lacticaseibacillus rhamnosus* MP108.	Oral.	SCORAD, IDQLQ.	Probiotic was effective in reducing symptoms of AD after 8 weeks of treatment.
Ibañez et al. [139]/Spain	*N* = 320 children (average age: 5.1). Eight weeks. Observational prospective study.	Synbiotic: *Lacticaseibacilluscasei* LC5, *L. rhamnosus* LR5, *Lactiplantibacillus plantarum* LP3, and *Bifidobacterium lactis* BL3 (probiotics). Fructooligosaccharides (FOS), galactooligosaccharides (GOS), with biotin (prebiotics).	Oral.	SCORAD, VAS.	SCORAD index and VAS score for pruritus decreased after synbiotic treatment.
Myles et al. [140]/USA	*N* = 15 AD patients (10 adults and 5 children). 10 weeks. Open label phase I.	Topical microbiome transplantation: *Roseomonas mucosa*.	Topic.	SCORAD.	Treatment with *R. mucosa* was associated with significant decreases in measures of disease severity. There were no adverse events on treatment application.
Navarro-López et al. [141]/Spain	*N* = 25 AD patients (age: 4–17; average age: 9.3). 12 weeks. Double-blind, placebo-controlled study.	Probiotics: *Bifidobacterium animalis* subsp. *lactis* CECT 8145, *B. longum* CECT 7347 and *L. casei* CECT 9104.	Oral.	SCORAD.	The mixture of probiotics was effective in reducing SCORAD index and in reducing the use of topical steroids in patients with moderate AD.
Dissanayake et al. [142]/Japan	*N* = 605 pregnant women (age: 24–32) and 549 babies (age: 0–6 months). Follow-up: 4 years. 2 × 2 factorial randomized controlled trial.	Synbiotic: *Bifibobacterium bifidum* OLB6378 (probiotic), with FOS (prebiotic).	Oral.	EASI.	Neither skin care nor the synbiotic showed any effect on reducing the development of AD and food allergens at 1 year of age.
Ahn et al. [143]/Korea	*N* = 124 AD patients (age:2–13). Twelve weeks. Double-blinded, placebo controlled randomized study.	Probiotic: *Lactiplantibacillus pentosus*.	Oral.	SCORAD.	Improved symptoms were recorded both in the probiotic and placebo groups, but SCORAD index for the probiotic group was significantly improved compared to those for the placebo group in allergen-sensitized AD.
Climent et al. [144]/Spain	*N* = 50 AD patients (age: 4–17). Twelve weeks. Double-blind, placebo controlled randomized study.	Probiotics: *B. animalis* subsp. *lactis* CECT 8145, *B. longum* CECT 7347 and *L. casei* CECT 9104.	Oral.	SCORAD.	Probiotic mixture treatment showed a significant improvement in SCORAD index. The treatment modulated the gut microbiome with significant changes in the genera *Faecalibacterium* and *Bacteroides*.
Noll et al. [145]/Germany	*N* = 22 AD patients. Fourteen days. Three bath groups (synbiotic, prebiotic, and control). Double-blind, randomized study.	Synbiotic: *Bifibobacterium breve* ATCC 15698, *B. animalis* subsp. *lactis* ATCC 27536, *L. casei* ATCC 393, *L. plantarum* ATCC 14917, *L. rhamnosus* ATCC 53103, and *Lactobacillus gasseri* ATCC 33323 (probiotics), with maltodextrin, inulin, and apple pectin (prebiotics).	Topic.	SCORAD, QoL.	Significantly reduced SCORAD over time of AD patients after daily synbiotic or prebiotic baths. Synbiotic baths improved pruritus and skin dryness. Improved QoL indices.
Carucci et al. [146]/Italy	*N* = 100 AD patients (age: 6–36 months). Twelve weeks. Double-blind, randomized controlled study.	Probiotic: *L. rhamnosus* GG.	Oral.	SCORAD, IDQoL, ProPAD.	Beneficial effects on disease severity and quality of life were obtained with the probiotic treatment.
De Andrade et al. [147]/Brazil	*N* = 60 AD patients (age: 6 months–19 years). Six–twelve months. Double-blind, randomized, placebo-controlled clinical trial.	Probiotics: *B. animalis* subsp. *lactis* HN019, *L. rhamnosus* HN001, *Lacticaseibacillus paracasei* Lep57, and *Lactobacillus acidophilus* NCFM.	Oral.	SCORAD.	Children and adolescent with AD presented a significant positive clinical response after 6 months with the probiotic cocktail treatment.
Wang et al. [148]/Hong Kong-China	*N* = 41 AD patients (age: 18–73; average age: 47). Eight weeks. Pilot study.	Mixture of probiotics, prebiotics and postbiotics (E3 preparation): *B. animalis* subsp. *lactis* GKK2, *B. bifidum* GKB2, *L. rhamnosus* GG, *L. paracasei* GK56, *L. acidophilus* GK47, *L. casei* GKC1, and *Lactobacillus lactis* subsp. *lactis* GKL2 (probiotics), with FOS, GOS, inulin (prebiotics), and with heat-inactivated *L. plantarum* (postbiotic).	Oral.	EASI.	EASI of the participants was significantly lower after the E3 treatment.
Colombo et al. [149]/Italy	*N* = 144 AD patients (average age: 25.1). Twelve weeks. Multicenter, retrospective observational study.	Synbiotic: *B. animalis* subsp. *lactis* BSO1, *L. plantarum* LP14, and *L. rhamnosus* LR05 (probiotics), with FOS and vitamin B2 (prebiotics).	Oral.	SCORAD, EASI, TIS.	Pruritus and AD-related lesions (erythema, edema, papules, and excoriation) exhibited significant clinical improvement.

CDLQI: Children’s Dermatology Life Quality Index; EASI: Eczema Area and Severity Index; FDLQI: Family Dermatology Life Quality Index; IDQLD: Infant Dermatitis Quality of Life Questionnaire; IDQoL: Infant Dermatitis Quality of Life Questionnaire; ProPAD: Probiotic for Pediatric Atopic Dermatitis; QoL: Quality of Life Index; SCORAD: Scoring Atopic Dermatitis; TIS: Three-Item Severity; VAS: Visual Analogue Score.

## References

[1] Lal M.K., Sharma E., Tiwari R.K., Devi R., Mishra U.N., Thakur R., Gupta R., Dey A., Lal P., Sahu S.K. (2022). Nutrient-mediated perception and signalling in human metabolism: A perspective of nutrigenomics. Int. J. Mol. Sci..

[2] Borrego-Ruiz A., Borrego J.J. (2024). Human gut microbiome, diet, and mental disorders. Int. Microbiol..

[3] Flint H.J. (2012). The impact of nutrition on the human microbiome. Nutr. Rev..

[4] Mahmud M.R., Akter S., Tamanna S.K., Mazumder L., Esti I.Z., Banerjee S., Akter S., Hasan M.R., Acharjee M., Hossain M.S. (2022). Impact of gut microbiome on skin health: Gut-skin axis observed through the lenses of therapeutics and skin diseases. Gut Microbes.

[5] De Pessemier B., Grine L., Debaere M., Maes A., Paetzold B., Callewaert C. (2021). Gut–skin axis: Current knowledge of the interrelationship between microbial dysbiosis and skin conditions. Microorganisms.

[6] Glatthardt T., Lima R.D., de Mattos R.M., Ferreira R.B.R. (2024). Microbe interactions within the skin microbiome. Antibiotics.

[7] Townsend E.C., Kalan L.R. (2023). The dynamic balance of the skin microbiome across the lifespan. Biochem. Soc. Trans..

[8] Cundell A.M. (2018). Microbial ecology of the human skin. Microb. Ecol..

[9] Nguyen U.T., Kalan L.R. (2022). Forgotten fungi: The importance of the skin mycobiome. Curr. Opin. Microbiol..

[10] Hannigan G.D., Meisel J.S., Tyldsley A.S., Zheng Q., Hodkinson B.P., SanMiguel A.J., Minot S., Bushman F.D., Grice E.A. (2015). The human skin double-stranded DNA virome: Topographical and temporal diversity, genetic enrichment, and dynamic associations with the host microbiome. mBio.

[11] Natarelli N., Gahoonia N., Sivamani R.K. (2023). Bacteriophages and the microbiome in dermatology: The role of the phageome and a potential therapeutic strategy. Int. J. Mol. Sci..

[12] Chen Y.E., Fischbach M.A., Belkaid Y. (2018). Skin microbiota-host interactions. Nature.

[13] Carmona-Cruz S., Orozco-Covarrubias L., Sáez de Ocariz M. (2022). The human skin microbiome in selected cutaneous diseases. Front. Cell. Infect. Microbiol..

[14] Bowe W.P., Logan A.C. (2011). Acne vulgaris, probiotics and the gut-brain-skin axis—Back to the future?. Gut Pathog..

[15] Shah K.R., Boland C.R., Patel M., Thrash B., Menter A. (2013). Cutaneous manifestations of gastrointestinal disease: Part I. J. Am. Acad. Dermatol..

[16] Thrash B., Patel M., Shah K.R. (2013). Cutaneous manifestations of gastrointestinal disease: Part II. J. Am. Acad. Dermatol..

[17] Guet-Revillet H., Jais J.P., Ungeheuer M.N., Coignard-Biehler H., Duchatelet S., Delage M., Lam T., Hovnanian A., Lortholary O., Nassif X. (2017). The microbiological landscape of anaerobic infections in hidradenitis suppurativa: A prospective metagenomic study. Clin. Infect. Dis..

[18] O’Neill C.A., Monteleone G., McLaughlin J., Paus R. (2016). The gut-skin axis in health and disease: A paradigm with therapeutic implications. BioEssays.

[19] Vaughn A.R., Notay M., Clark A.K., Sivamani R.K. (2017). Skin-gut axis: The relationship between intestinal bacteria and skin health. World J. Dermatol..

[20] Cianciulli A., Calvello R., Porro C., Lofrumento D.D., Panaro M.A. (2024). Inflammatory skin diseases: Focus on the role of suppressors of cytokine signaling (SOCS) proteins. Cells.

[21] Baba H., Masuyama A., Yoshimura C., Aoyama Y., Takano T., Ohki K. (2010). Oral intake of *Lactobacillus helveticus*-fermented milk whey decreased transepidermal water loss and prevented the onset of sodium dodecylsulfate-induced dermatitis in mice. Biosci. Biotechnol. Biochem..

[22] Philippe D., Blum S., Benyacoub J. (2011). Oral *Lactobacillus paracasei* improves skin barrier function recovery and reduces local skin inflammation. Eur. J. Dermatol..

[23] Gueniche A., Philippe D., Bastien P., Reuteler G., Blum S., Castiel-Higounenc I., Breton L., Benyacoub J. (2014). Randomised double-blind placebo-controlled study of the effect of *Lactobacillus paracasei* NCC 2461 on skin reactivity. Benef. Microbes.

[24] Borrego-Ruiz A., Borrego J.J. (2024). An updated overview on the relationship between human gut microbiome dysbiosis and psychiatric and psychological disorders. Prog. Neuropsychopharmacol. Biol. Psychiatry.

[25] Forbes J.D., Van Domselaar G., Bernstein C.N. (2016). The gut microbiota in immune-mediated inflammatory diseases. Front. Microbiol..

[26] Salem I., Ramser A., Isham N., Ghannoum M.A. (2018). The gut microbiome as a major regulator of the gut-skin axis. Front. Microbiol..

[27] Schwarz A., Bruhs A., Schwarz T. (2017). The short-chain fatty acid sodium butyrate functions as a regulator of the skin immune system. J. Investig. Dermatol..

[28] Gallitano S.M., Berson D.S. (2018). How acne bumps cause the blues: The influence of acne vulgaris on self-esteem. Int. J. Womens Dermatol..

[29] Morshed A.S.M., Noor T., Uddin Ahmed M.A., Mili F.S., Ikram S., Rahman M., Ahmed S., Uddin M.B. (2023). Understanding the impact of acne vulgaris and associated psychological distress on self-esteem and quality of life via regression modeling with CADI, DLQI, and WHOQoL. Sci. Rep..

[30] Vasam M., Korutla S., Bohara R.A. (2023). Acne vulgaris: A review of the pathophysiology, treatment, and recent nanotechnology based advances. Biochem. Biophys. Rep..

[31] Deng Y., Wang H., Zhou J., Mou Y., Wang G., Xiong X. (2018). Patients with acne vulgaris have a distinct gut microbiota in comparison with healthy controls. Acta Derm. Venereol..

[32] Yan H.M., Zhao H.J., Guo D.Y., Zhu P.Q., Zhang C.L., Jiang W. (2018). Gut microbiota alterations in moderate to severe acne vulgaris patients. J. Dermatol..

[33] Thompson K.G., Rainer B.M., Antonescu C., Florea L., Mongodin E.F., Kang S., Chien A.L. (2020). Minocycline and its impact on microbial dysbiosis in the skin and gastrointestinal tract of acne patients. Ann. Dermatol..

[34] Huang Y., Liu L., Chen L., Zhou L., Xiong X., Deng Y. (2021). Gender-specific differences in gut microbiota composition associated with microbial metabolites for patients with acne vulgaris. Ann. Dermatol..

[35] Moreno-Arrones O.M., Serrano-Villar S., Perez-Brocal V., Saceda-Corralo D., Morales-Raya C., Rodrigues-Barata R., Moya A., Jaen-Olasolo P., Vano-Galvan S. (2020). Analysis of the gut microbiota in alopecia areata: Identification of bacterial biomarkers. J. Eur. Acad. Dermatol. Venereol..

[36] Juhasz M., Chen S., Khosrovi-Eghbal A., Ekelem C., Landaverde Y., Baldi P., Mesinkovska N.A. (2020). Characterizing the skin and gut microbiome of alopecia areata patients. SKIN J. Cutan. Med..

[37] Brzychcy K., Dróżdż I., Skoczylas S., Płoszaj T., Sobolewska-Sztychny D., Skibińska M., Narbutt J., Lesiak A. (2022). Gut microbiota in alopecia areata. Postep. Derm. Alergol..

[38] Song H., Yoo Y., Hwang J., Na Y.C., Kim H.S. (2016). *Faecalibacterium prausnitzii* subspecies-level dysbiosis in the human gut microbiome underlying atopic dermatitis. J. Allergy Clin. Immunol..

[39] Nylund L., Nermes M., Isolauri E., Salminen S., de Vos W.M., Satokari R. (2015). Severity of atopic disease inversely correlates with intestinal microbiota diversity and butyrate-producing bacteria. Allergy.

[40] Lee E., Lee S.Y., Kang M.J., Kim K., Won S., Kim B.J., Choi K.Y., Kim B.S., Cho H.J., Kim Y. (2016). Clostridia in the gut and onset of atopic dermatitis via eosinophilic inflammation. Ann. Allergy Asthma Immunol..

[41] Reddel S., Del Chierico F., Quagliariello A., Giancristoforo S., Vernocchi P., Russo A., Fiocchi A., Rossi P., Putignani L., El Hachem M. (2019). Gut microbiota profile in children affected by atopic dermatitis and evaluation of intestinal persistence of a probiotic mixture. Sci. Rep..

[42] Park Y.M., Lee S.Y., Kang M.J., Kim B.S., Lee M.J., Jung S.S., Yoon J.S., Cho H.J., Lee E., Yang S.I. (2020). Imbalance of gut *Streptococcus*, *Clostridium*, and *Akkermansia* determines the natural course of atopic dermatitis in infant. Allergy Asthma Immunol. Res..

[43] Nekrasova A.I., Kalashnikova I.G., Bobrova M.M., Korobeinikova A.V., Bakoev S.Y., Ashniev G.A., Petryaikina E.S., Nekrasov A.S., Zagainova A.V., Lukashina M.V. (2024). Characteristics of the gut microbiota in regard to atopic dermatitis and food allergies of children. Biomedicines.

[44] Ryguła I., Pikiewicz W., Kaminiów K. (2024). Impact of diet and nutrition in patients with acne vulgaris. Nutrients.

[45] Melnik B.C. (2015). Linking diet to acne metabolomics, inflammation, and comedogenesis: An update. Clin. Cosmet. Investig. Dermatol..

[46] Mounessa J., Caravaglio J.V., Domozych R., Chapman S., Dellavalle R.P., Dunnick C.A., Norris D. (2023). Commonly prescribed medications associated with alopecia. J. Am. Acad. Dermatol..

[47] Oiwoh S.O., Enitan A.O., Adegbosin O.T., Akinboro A.O., Onayemi E.O. (2024). Androgenetic alopecia: A review. Niger. Postgrad. Med. J..

[48] Sibbald C. (2023). Alopecia areata: An updated review for 2023. J. Cutan. Med. Surg..

[49] Nair L., Dai Z., Christiano A.M. (2017). Gut microbiota plays a role in the development of alopecia areata. J. Investig. Dermatol..

[50] Rebello D., Wang E., Yen E., Lio P.A., Kelly C.R. (2017). Hair growth in two alopecia patients after fecal microbiota transplant. ACG Case Rep. J..

[51] Simakou T., Butcher J.P., Reid S., Henriquez F.L. (2019). Alopecia areata: A multifactorial autoimmune condition. J. Autoimmun..

[52] Migacz-Gruszka K., Branicki W., Obtulowicz A., Pirowska M., Gruszka K., Wojas-Pelc A. (2019). What’s new in the pathophysiology of alopecia areata? The possible contribution of skin and gut microbiome in the pathogenesis of alopecia—Big opportunities, big challenges, and novel perspectives. Int. J. Trichology.

[53] Garg S., Sangwan A. (2019). Dietary protein deficit and deregulated autophagy: A new clinico-diagnostic perspective in pathogenesis of early aging, skin, and hair disorders. Indian Dermatol. Online J..

[54] Pham C.T., Romero K., Almohanna H.M., Griggs J., Ahmed A., Tosti A. (2020). The role of diet as an adjuvant treatment in scarring and nonscarring alopecia. Skin Appendage Disord..

[55] Afshari M., Kolackova M., Rosecka M., Čelakovská J., Krejsek J. (2024). Unraveling the skin; a comprehensive review of atopic dermatitis, current understanding, and approaches. Front. Immunol..

[56] Lönndahl L., Abdelhadi S., Holst M., Lonne-Rahm S.B., Nordlind K., Johansson B. (2023). Psychological stress and atopic dermatitis: A focus group study. Ann. Dermatol..

[57] Sandhu J.K., Wu K.K., Bui T.L., Armstrong A.W. (2019). Association between atopic dermatitis and suicidality: A systematic review and meta-analysis. JAMA Dermatol..

[58] Hu C., van Meel E.R., Medina-Gomez C., Kraaij R., Barroso M., Kiefte-de Jong J., Radjabzadeh D., Pasmans S., de Jong N.W., de Jongste J.C. (2021). A population-based study on associations of stool microbiota with atopic diseases in school-age children. J. Allergy Clin. Immunol..

[59] Lee S.Y., Lee E., Park Y.M., Hong S.J. (2018). Microbiome in the gut-skin axis in atopic dermatitis. Allergy Asthma Immunol. Res..

[60] Fang Z., Li L., Zhang H., Zhao J., Lu W., Chen W. (2021). Gut microbiota, probiotics, and their interactions in prevention and treatment of atopic dermatitis: A review. Front. Immunol..

[61] Belkaid Y., Hand T.W. (2014). Role of the microbiota in immunity and inflammation. Cell.

[62] Moniaga C.S., Tominaga M., Takamori K. (2022). An altered skin and gut microbiota are involved in the modulation of itch in atopic dermatitis. Cells.

[63] Kim J., Kim B.E., Leung D.Y.M. (2019). Pathophysiology of atopic dermatitis: Clinical implications. Allergy Asthma Proc..

[64] Guo X., Li J., Tang R., Zhang G., Zeng H., Wood R.J., Liu Z. (2017). High fat diet alters gut microbiota and the expression of Paneth cell-antimicrobial peptides preceding changes of circulating inflammatory cytokines. Mediators Inflamm..

[65] Devereux G., Seaton A. (2005). Diet as a risk factor for atopy and asthma. J. Allergy Clin. Immunol..

[66] Sharma N., Chaudhary S.M., Khungar N., Aulakh S.K., Idris H., Singh A., Sharma K. (2024). Dietary influences on skin health in common dermatological disorders. Cureus.

[67] Borrego-Ruiz A. (2024). Una revisión crítica sobre la influencia de la dieta vegetariana en la salud mental. Rev. Esp. Nutr. Comun..

[68] Baldwin H., Tan J. (2021). Effects of diet on acne and its response to treatment. Am. J. Clin. Dermatol..

[69] Stewart T.J., Bazergy C. (2018). Hormonal and dietary factors in acne vulgaris versus controls. Dermatoendocrinol..

[70] Dréno B. (2017). What is new in the pathophysiology of acne, an overview. J. Eur. Acad. Dermatol. Venereol..

[71] Flores-Balderas X., Peña-Peña M., Rada K.M., Alvarez-Alvarez Y.Q., Guzmán-Martín C.A., Sánchez-Gloria J.L., Huang F., Ruiz-Ojeda D., Morán-Ramos S., Springall R. (2023). Beneficial effects of plant-based diets on skin health and inflammatory skin diseases. Nutrients.

[72] Suppiah T.S.S., Sundram T.K.M., Tan E.S.S., Lee C.K., Bustami N.A., Tan C.K. (2018). Acne vulgaris and its association with dietary intake: A Malaysian perspective. Asia Pac. J. Clin. Nutr..

[73] Aalemi A.K., Anwar I., Chen H. (2019). Dairy consumption and acne: A case control study in Kabul, Afghanistan. Clin. Cosmet. Investig. Dermatol..

[74] Sanchez-Pellicer P., Navarro-Moratalla L., Núñez-Delegido E., Ruzafa-Costas B., Agüera-Santos J., Navarro-López V. (2022). Acne, microbiome, and probiotics: The gut-skin axis. Microorganisms.

[75] Meixiong J., Ricco C., Vasavda C., Ho B.K. (2022). Diet and acne: A systematic review. JAAD Int..

[76] Fusano M. (2023). Veganism in acne, atopic dermatitis, and psoriasis: Benefits of a plant-based diet. Clin. Dermatol..

[77] Rao A., Douglas S.C., Hall J.M. (2021). Endocrine disrupting chemicals, hormone receptors, and acne vulgaris: A connecting hypothesis. Cells.

[78] Riyanto P., Subchan P., Lelyana R. (2015). Advantage of soybean isoflavone as antiandrogen on acne vulgaris. Dermatoendocrinology.

[79] Lee H., Sim N., Fotouhi A., Daveluy S. (2023). Vegan diet in dermatology: A review. J. Clin. Med..

[80] Neufingerl N., Eilander A. (2021). Nutrient intake and status in adults consuming plant-based fiets compared to meat-eaters: A systematic review. Nutrients.

[81] Guo E.L., Katta R. (2017). Diet and hair loss: Effects of nutrient deficiency and supplement use. Dermatol. Pract. Concept..

[82] Lai C.H., Chu N.F., Chang C.W., Wang S.L., Yang H.C., Chu C.M., Chang C.T., Lin M.H., Chien W.C., Su S.L. (2013). Androgenic alopecia is associated with less dietary soy, lower blood vanadium and rs1160312 1 polymorphism in Taiwanese communities. PLoS ONE.

[83] English R.S., Barazesh J.M. (2019). Self-assessments of standardized scalp massages for androgenic alopecia: Survey results. Dermatol. Ther..

[84] Greenberg S.A. (2020). Diet and skin: A primer. Cutis.

[85] Kouda K., Tanaka T., Kouda M., Takeuchi H., Takeuchi A., Nakamura H., Takigawa M. (2000). Low-energy diet in atopic dermatitis patients: Clinical findings and DNA damage. J. Physiol. Anthropol. Appl. Hum. Sci..

[86] Tanaka T., Kouda K., Kotani M., Takeuchi A., Tabei T., Masamoto Y., Nakamura H., Takigawa M., Suemura M., Takeuchi H. (2001). Vegetarian diet ameliorates symptoms of atopic dermatitis through reduction of the number of peripheral eosinophils and of PGE2 synthesis by monocytes. J. Physiol. Anthropol. Appl. Hum. Sci..

[87] Zhang J., Loman L., Oldhoff M., Schuttelaar M.L.A. (2022). Association between moderate to severe atopic dermatitis and lifestyle factors in the Dutch general population. Clin. Exp. Dermatol..

[88] Sakkas H., Bozidis P., Touzios C., Kolios D., Athanasiou G., Athanasopoulou E., Gerou I., Gartzonika C. (2020). Nutritional status and the influence of the vegan diet on the gut microbiota and human health. Medicina.

[89] Tomova A., Bukovsky I., Rembert E., Yonas W., Alwarith J., Barnard N.D., Kahleova H. (2019). The effects of vegetarian and vegan diets on gut microbiota. Front. Nutr..

[90] Borrego-Ruiz A., Borrego J.J. (2024). Influence of the vegetarian diet on the human intestinal microbiome [Influencia de la dieta vegetariana en el microbioma intestinal humano]. Nutr. Clín. Diet. Hosp..

[91] Khan A., Adalsteinsson J., Whitaker-Worth D.L. (2022). Atopic dermatitis and nutrition. Clin. Dermatol..

[92] Figueroa C., Echeverría G., Villarreal G., Martínez X., Ferreccio C., Rigotti A. (2021). Introducing plant-based Mediterranean diet as a lifestyle medicine approach in Latin America: Opportunities within the Chilean context. Front. Nutr..

[93] Mansilla-Polo M., Piquero-Casals J., Morgado-Carrasco D. (2024). Popular diets and skin effects: A narrative review [Dietas populares y su impacto en la piel. Una revisión narrativa]. Actas Dermosifiliogr..

[94] Delgado-Lista J., Alcala-Diaz J.F., Torres-Peña J.D., Quintana Navarro G.M., Fuentes F., Garcia-Rios A., Ortiz-Morales A.M., Gonzalez-Requero A.I., Perez-Caballero A.I., Yubero-Serrano E.M. (2022). Long-term secondary prevention of cardiovascular disease with a Mediterranean diet and a low-fat diet (CORDIOPREV): A randomized controlled trial. Lancet.

[95] Kaluza J., Lozynska K., Rudzinska J., Granda D., Sicinska E., Szmidt M.K. (2023). Mediterranean-style diet and other determinants of well-being in omnivorous, vegetarian, and vegan women. Nutrients.

[96] Barrea L., Donnarumma M., Cacciapuoti S., Muscogiuri G., De Gregorio L., Blasio C., Savastano S., Colao A., Fabbrocini G. (2021). Phase angle and Mediterranean diet in patients with acne: Two easy tools for assessing the clinical severity of disease. J. Transl. Med..

[97] Ah-Thiane L., Nguyen J.M., Khammari A., Dréno B. (2022). Lifestyle habits and impact of the Mediterranean diet on facial acne severity in French women: A case-control study. Int. J. Womens Dermatol..

[98] Bertolani M., Rodighiero E., Saleri R., Pedrazzi G., Bertoli S., Leone A., Feliciani C., Lotti T., Satolli F. (2022). The influence of Mediterranean diet in acne pathogenesis and the correlation with insulin-like growth factor-1 serum levels: Implications and results. Dermatol. Rep..

[99] Wei G., Martirosyan D. (2019). Hair loss: A review of the role of food bioactive compounds. Bioact. Compod. Health Dis..

[100] Fortes C., Mastroeni S., Mannooranparampil T., Abeni D., Panebianco A. (2018). Mediterranean diet: Fresh herbs and fresh vegetables decrease the risk of androgenetic alopecia in males. Arch. Dermatol. Res..

[101] Antonogeorgos G., Mandrapylia M., Liakou E., Koutsokera A., Drakontaeidis P., Thanasia M., Ellwood P., García-Marcos L., Sardeli O., Priftis K.N. (2022). Hierarchical analysis of Mediterranean dietary pattern on atopic diseases’ prevalence in adolescence: The Greek Global Asthma Network study. Allergol. Immunopathol..

[102] Bédard A., Northstone K., Henderson A.J., Shaheen S.O. (2020). Mediterranean diet during pregnancy and childhood respiratory and atopic outcomes: Birth cohort study. Eur. Respir. J..

[103] Hill C., Guarner F., Reid G., Gibson G.R., Merenstein D.J., Pot B., Morelli L., Canani R.B., Flint H.J., Salminen S. (2014). Expert consensus document. The International Scientific Association for Probiotics and Prebiotics consensus statement on the scope and appropriate use of the term probiotic. Nat. Rev. Gastroenterol. Hepatol..

[104] Swanson K.S., Gibson G.R., Hutkins R., Reimer R.A., Reid G., Verbeke K., Scott K.P., Holscher H.D., Azad M.B., Delzenne N.M. (2020). The International Scientific Association for Probiotics and Prebiotics (ISAPP) consensus statement on the definition and scope of synbiotics. Nat. Rev. Gastroenterol. Hepatol..

[105] Salminen S., Collado M.C., Endo A., Hill C., Lebeer S., Quigley E.M.M., Sanders M.E., Shamir R., Swann J.R., Szajewska H. (2021). The International Scientific Association of Probiotics and Prebiotics (ISAPP) consensus statement on the definition and scope of postbiotics. Nat. Rev. Gastroenterol. Hepatol..

[106] Al-Ghazzewi F., Tester R. (2014). Impact of prebiotics and probiotics on skin health. Benef. Microbes.

[107] Lizardo M.P., Tavaria F.K. (2022). Probiotic growth in skin-like conditions. AIMS Microbiol..

[108] Nakatsuji T., Chen T.H., Narala S., Chun K.A., Two A.M., Yun T., Shafiq F., Kotol P.F., Bouslimani A., Melnik A.V. (2017). Antimicrobials from human skin commensal bacteria protect against *Staphylococcus aureus* and are deficient in atopic dermatitis. Sci. Transl. Med..

[109] Lizardo M., Magalhães R.M., Tavaria F.K. (2022). Probiotic adhesion to skin keratinocytes and underlying mechanisms. Biology.

[110] Paetzold B., Willis J.R., Pereira de Lima J., Knödlseder N., Brüggemann H., Quist S.R., Gabaldón T., Güell M. (2019). Skin microbiome modulation induced by probiotic solutions. Microbiome.

[111] Abdi A., Oroojzadeh P., Valivand N., Sambrani R., Lotfi H. (2024). Immunological aspects of probiotics for improving skin diseases: Influence on the gut-brain-skin axis. Biochem. Biophys. Res. Commun..

[112] Benyacoub J., Bosco N., Blanchard C., Demont A., Philippe D., Castiel-Higounenc I., Guéniche A. (2014). Immune modulation property of *Lactobacillus paracasei* NCC2461 (ST11) strain and impact on skin defences. Benef. Microbes.

[113] Poutahidis T., Kearney S.M., Levkovich T., Qi P., Varian B.J., Lakritz J.R., Ibrahim Y.M., Chatzigiagkos A., Alm E.J., Erdman S.E. (2013). Microbial symbionts accelerate wound healing via the neuropeptide hormone oxytocin. PLoS ONE.

[114] Ghoshal U.C., Shukla R., Ghoshal U. (2017). Small intestinal bacterial overgrowth and irritable bowel syndrome: A bridge between functional organic dichotomy. Gut Liver.

[115] Roszkowska P., Klimczak E., Ostrycharz E., Rączka A., Wojciechowska-Koszko I., Dybus A., Cheng Y.H., Yu Y.H., Mazgaj S., Hukowska-Szematowicz B. (2024). Small intestinal bacterial overgrowth (SIBO) and twelve groups of related diseases-current state of knowledge. Biomedicines.

[116] Wang F.Y., Chi C.C. (2021). Rosacea, germs, and bowels: A review on gastrointestinal comorbidities and gut-skin axis of rosacea. Adv. Ther..

[117] Kim J., Ko Y., Park Y.K., Kim N.I., Ha W.K., Cho Y. (2010). Dietary effect of lactoferrin-enriched fermented milk on skin surface lipid and clinical improvement of acne vulgaris. Nutrition.

[118] Colletti A., Pellizzato M., Cicero A.F. (2023). The possible role of probiotic supplementation in inflammation: A narrative review. Microorganisms.

[119] Jung G.W., Tse J.E., Guiha I., Rao J. (2013). Prospective, randomized, open-label trial comparing the safety, efficacy, and tolerability of an acne treatment regimen with and without a probiotic supplement and minocycline in subjects with mild to moderate acne. J. Cutan. Med. Surg..

[120] Fabbrocini G., Bertona M., Picazo Ó., Pareja-Galeano H., Monfrecola G., Emanuele E. (2016). Supplementation with *Lactobacillus rhamnosus* SP1 normalises skin expression of genes implicated in insulin signalling and improves adult acne. Benef. Microbes.

[121] Majeed M., Majeed S., Nagabhushanam K., Mundkur L., Rajalakshmi H.R., Shah K., Beede K. (2020). Novel topical application of a postbiotic, LactoSporin^®^, in mild to moderate acne: A randomized, comparative clinical study to evaluate its efficacy, tolerability and safety. Cosmetics.

[122] Cui H., Guo C., Wang Q., Feng C., Duan Z. (2022). A pilot study on the efficacy of topical lotion containing anti-acne postbiotic in subjects with mild -to -moderate acne. Front. Med..

[123] Ma’or Z., Temmerman R., Zhang X. (2023). Topical application of synbiotic *Bacillus* preparations positively affects skin (micro) biology. J. Cosmet. Dermatol. Sci. Appl..

[124] Podrini C., Schramm L., Marianantoni G., Apolinarska J., McGuckin C., Forraz N., Milet C., Desroches A.L., Payen P., D’Aguanno M. (2023). Topical administration of *Lactiplantibacillus plantarum* (SkinDuo^TM^) serum improves anti-acne properties. Microorganisms.

[125] Rybak I., Haas K.N., Dhaliwal S.K., Burney W.A., Pourang A., Sandhu S.S., Maloh J., Newman J.W., Crawford R., Sivamani R.K. (2023). Prospective placebo-controlled assessment of spore-based probiotic supplementation on sebum production, skin barrier function, and acne. J. Clin. Med..

[126] Levkovich T., Poutahidis T., Smillie C., Varian B.J., Ibrahim Y.M., Lakritz J.R., Alm E.J., Erdman S.E. (2013). Probiotic bacteria induce a ‘glow of health’. PLoS ONE.

[127] Horii Y., Kaneda H., Fujisaki Y., Fuyuki R., Nakakita Y., Shigyo T., Nagai K. (2014). Effect of heat-killed *Lactobacillus brevis* SBC8803 on cutaneous arterial sympathetic nerve activity, cutaneous blood flow and transepidermal water loss in rats. J. Appl. Microbiol..

[128] Ogawa M., Saiki A., Matsui Y., Tsuchimoto N., Nakakita Y., Takata Y., Nakamura T. (2016). Effects of oral intake of heat-killed *Lactobacillus brevis* SBC8803 (SBL88™) on dry skin conditions: A randomized, double-blind, placebo-controlled study. Exp. Ther. Med..

[129] Xie W.R., Yang X.Y., Xia H.H., Wu L.H., He X.X. (2019). Hair regrowth following fecal microbiota transplantation in an elderly patient with alopecia areata: A case report and review of the literature. World J. Clin. Cases.

[130] Park D.W., Lee H.S., Shim M.S., Yum K.J., Seo J.T. (2020). Do Kimchi and *Cheonggukjang* probiotics as a functional food improve androgenetic alopecia? A clinical pilot study. World J. Mens Health.

[131] Rinaldi F., Trink A., Pinto D. (2020). Efficacy of postbiotics in a PRP-like cosmetic product for the treatment of alopecia area celsi: A randomized double-blinded parallel-group study. Dermatol. Ther..

[132] Liang C.H., Lin Y.H., Chan S.T., Lin Y.K., Chiang C.F. (2022). *Lactiplantibacillus plantarum* TCI999 probiotic promoted hair growth and regulated gut microbiome: Double-blind, placebo-controlled trial. J. Probiot. Health.

[133] Navarro-Belmonte M.R., Aguado-García Á., Sánchez-Pellicer P., Núñez-Delegido E., Navarro-Moratalla L., Martínez-Villaescusa M., García-Navarro A., Navarro-López V. (2024). The effect of an oral probiotic mixture on clinical evolution and the gut and skin microbiome in patients with alopecia areata: A randomized clinical trial. Cosmetics.

[134] Rusu E., Enache G., Cursaru R., Alexescu A., Radu R., Onila O., Cavallioti T., Rusu F., Posea M., Jinga M. (2019). Prebiotics and probiotics in atopic dermatitis. Exp. Ther. Med..

[135] Kim H.J., Kim H.Y., Lee S.Y., Seo J.H., Lee E., Hong S.J. (2013). Clinical efficacy and mechanism of probiotics in allergic diseases. Korean J. Pediatr..

[136] Wang I.J., Wang J.Y. (2015). Children with atopic dermatitis show clinical improvement after *Lactobacillus* exposure. Clin. Exp. Allergy.

[137] Blanchet-Réthoré S., Bourdès V., Mercenier A., Haddar C.H., Verhoeven P.O., Andres P. (2017). Effect of a lotion containing the heat-treated probiotic strain *Lactobacillus johnsonii* NCC 533 on *Staphylococcus aureus* colonization in atopic dermatitis. Clin. Cosmet. Investigat. Dermatol..

[138] Wu Y.J., Wu W.F., Hung C.W., Ku M.S., Liao P.F., Sun H.L., Lu K.H., Sheu J.N., Lue K.H. (2017). Evaluation of efficacy and safety of *Lactobacillus rhamnosus* in children aged 4-48 months with atopic dermatitis: An 8-week, double-blind, randomized, placebo-controlled study. J. Microbiol. Immunol. Infect..

[139] Ibáñez M.D., Rodríguez Del Río P., González-Segura Alsina D., Villegas Iglesias V. (2018). Effect of synbiotic supplementation on children with atopic dermatitis: An observational prospective study. Eur. J. Pediatr..

[140] Myles I.A., Earland N.J., Anderson E.D., Moore I.N., Kieh M.D., Williams K.W., Saleem A., Fontecilla N.M., Welch P.A., Darnell D.A. (2018). First-in-human topical microbiome transplantation with *Roseomonas mucosa* for atopic dermatitis. JCI Insight.

[141] Navarro-López V., Ramírez-Boscá A., Ramón-Vidal D., Ruzafa-Costas B., Genovés-Martínez S., Chenoll-Cuadros E., Carrión-Gutiérrez M., Horga de la Parte J., Prieto-Merino D., Codoñer-Cortés F.M. (2018). Effect of oral administration of a mixture of probiotic strains on SCORAD index and use of topical steroids in young patients with moderate atopic dermatitis: A randomized clinical trial. JAMA Dermatol..

[142] Dissanayake E., Tani Y., Nagai K., Sahara M., Mitsuishi C., Togawa Y., Suzuki Y., Nakano T., Yamaide F., Ohno H. (2019). Skin care and synbiotics for prevention of atopic dermatitis or food allergy in newborn infants: A 2 × 2 factorial, randomized, non-treatment controlled trial. Int. Arch. Allergy Immunol..

[143] Ahn S.H., Yoon W., Lee S.Y., Shin H.S., Lim M.Y., Nam Y.D., Yoo Y. (2020). Effects of *Lactobacillus pentosus* in children with allergen-sensitized atopic dermatitis. J. Korean Med. Sci..

[144] Climent E., Martinez-Blanch J.F., Llobregat L., Ruzafa-Costas B., Carrión-Gutiérrez M.Á., Ramírez-Boscá A., Prieto-Merino D., Genovés S., Codoñer F.M., Ramón D. (2021). Changes in gut microbiota correlates with response to treatment with probiotics in patients with atopic dermatitis. A post hoc analysis of a clinical trial. Microorganisms.

[145] Noll M., Jäger M., Lux L., Buettner C., Axt-Gadermann M. (2021). Improvement of atopic dermatitis by synbiotic baths. Microorganisms.

[146] Carucci L., Nocerino R., Paparo L., De Filippis F., Coppola S., Giglio V., Cozzolino T., Valentino V., Sequino G., Bedogni G. (2022). Therapeutic effects elicited by the probiotic Lacticaseibacillus rhamnosus GG in children with atopic dermatitis. The results of the ProPAD trial. Pediatr. Allergy Immunol..

[147] de Andrade P.D.S.M.A., Maria E Silva J., Carregaro V., Sacramento L.A., Roberti L.R., Aragon D.C., Carmona F., Roxo-Junior P. (2022). Efficacy of probiotics in children and adolescents with atopic dermatitis: A randomized, double-blind, placebo-controlled study. Front. Nutr..

[148] Wang Y., Choy C.T., Lin Y., Wang L., Hou J., Tsui J.C.C., Zhou J., Wong C.H., Yim T.K., Tsui W.K. (2022). Effect of a novel E3 probiotics formula on the gut microbiome in atopic dermatitis patients: A pilot study. Biomedicines.

[149] Colombo D., Rigoni C., Cantù A., Carnevali A., Filippetti R., Franco T., Grassi A., Loi C., Mazzotta A., Patroi I. (2023). Probiotics and prebiotics orally assumed as disease modifiers for stable mild atopic dermatitis: An Italian real-life, multicenter, retrospective, observational study. Medicina.

[150] Yakupu A., Aimaier R., Yuan B., Chen B., Cheng J., Zhao Y., Peng Y., Dong J., Lu S. (2023). The burden of skin and subcutaneous diseases: Findings from the global burden of disease study 2019. Front. Public Health.

[151] Borrego-Ruiz A., Borrego J.J. (2024). Microbial dysbiosis in the skin microbiome and its psychological consequences. Microorganisms.

[152] Xiao X., Hu X., Yao J., Cao W., Zou Z., Wang L., Qin H., Zhong D., Li Y., Xue P. (2023). The role of short-chain fatty acids in inflammatory skin diseases. Front. Microbiol..

[153] Nagata H., Takagi N., Inoue S., Mizutani Y. (2021). Nicotine affects tight junction barriers via alpha 7 nicotine-like acetylcholine receptor in keratinocytes. J. Dermatol. Sci..

[154] Cevikbas F., Braz J.M., Wang X., Solorzano C., Sulk M., Buhl T., Steinhoff M., Basbaum A.I. (2017). Synergistic antipruritic effects of gamma aminobutyric acid A and B agonists in a mouse model of atopic dermatitis. J. Allergy Clin. Immunol..

[155] Langan E.A., Lisztes E., Bíró T., Funk W., Kloepper J.E., Griffiths C.E., Paus R. (2013). Dopamine is a novel, direct inducer of catagen in human scalp hair follicles in vitro. Br. J. Dermatol..

[156] Vaccaro R., Casini A., Severi C., Lamazza A., Pronio A., Palma R. (2023). Serotonin and melatonin in human lower gastrointestinal tract. Diagnostics.

[157] Baroni L., Rizzo G., Galchenko A.V., Zavoli M., Serventi L., Battino M. (2024). Health benefits of vegetarian diets: An insight into the main topics. Foods.

[158] Cena H., Calder P.C. (2020). Defining a healthy diet: Evidence for the role of contemporary dietary patterns in health and disease. Nutrients.

[159] Barrea L., Cacciapuoti S., Megna M., Verde L., Marasca C., Vono R., Camajani E., Colao A., Savastano S., Fabbrocini G. (2024). The effect of the ketogenic diet on acne: Could it be a therapeutic tool?. Crit. Rev. Food Sci. Nutr..

[160] Roster K., Xie L., Nguyen T., Lipner S.R. (2024). Impact of ketogenic and low-glycemic diets on inflammatory skin conditions. Cutis.

[161] Verde L., Frias-Toral E., Cacciapuoti S., Simancas-Racines D., Megna M., Caiazzo G., Potestio L., Maisto M., Tenore G.C., Colao A. (2024). Very low-calorie ketogenic diet (VLCKD): A therapeutic nutritional tool for acne?. J. Transl. Med..

[162] De Almeida C.V., Antiga E., Lulli M. (2023). Oral and topical probiotics and postbiotics in skincare and dermatological therapy: A concise review. Microorganisms.

[163] Suez J., Zmora N., Segal E., Elinav E. (2019). The pros, cons, and many unknowns of probiotics. Nat. Med..

[164] Lee G.R., Maarouf M., Hendricks A.J., Lee D.E., Shi V.Y. (2019). Topical probiotics: The unknowns behind their rising popularity. Dermatol. Online J..

[165] Ayichew T., Belete A., Alebachew T., Tsehaye H., Berhanu H., Minwuyelet A. (2017). Bacterial probiotics their importances and limitations: A review. J. Nutr. Health Sci..

[166] da Silva Vale A., de Melo Pereira G.V., de Oliveira A.C., de Carvalho Neto D.P., Herrmann L.W., Karp S.G., Soccol V.T., Soccol C.R. (2023). Production, formulation, and application of postbiotics in the treatment of skin conditions. Fermentation.

[167] Ming Z., Han L., Bao M., Zhu H., Qiang S., Xue S., Liu W. (2021). Living bacterial hydrogels for accelerated infected wound healing. Adv. Sci..

[168] Wu J.H., Cohen B.A. (2019). The stigma of skin disease. Curr. Opin. Pediatr..

[169] Chernyshov P.V., Tomas-Aragones L., Manolache L., Pustisek N., Darlenski R., Marron S.E., Koumaki D., Pochynok T.V., Szepietowski J.C., Wala-Zielinska K. (2024). Bullying in persons with skin diseases. J. Eur. Acad. Dermatol. Venereol..

[170] Borrego-Ruiz A., Fernández S. (2024). Humiliation and its relationship with bullying victimization: A narrative review. Psychol. Soc. Ed..

[171] Homayoon D., Hiebler-Ragger M., Zenker M., Weger W., Unterrainer H., Aberer E. (2020). Relationship between skin shame, psychological distress and quality of life in patients with psoriasis: A pilot study. Acta Derm. Venereol..

[172] Borrego-Ruiz A., Borrego J.J. (2024). Psychobiotics: A new perspective on the treatment of stress, anxiety, and depression. Anxiety Stress.

